# Status, evaluation, and influencing factors of biosecurity levels in pig farms in China

**DOI:** 10.1186/s12917-023-03827-6

**Published:** 2023-12-13

**Authors:** Jiamei Wang, Zizhong Shi, Xiangdong Hu

**Affiliations:** Institute of Agricultural Economics and Development, Chinese Academy of Agricultural Sciences, Beijing, 100081 China

**Keywords:** Biosecurity, Pig farms, China, Influencing factor, Rank sum ratio

## Abstract

**Background:**

Animal diseases have always been a serious threat to livestock breeding, and the establishment of a biosecurity barrier is important for disease prevention and control. Based on the investigations conducted in seven provinces located farms, this study aimed to explore the current biosecurity levels of farms in China, construct a biosecurity evaluation system, calculate the biosecurity levels of farms using the rank sum ratio comprehensive evaluation method, and develop an empirical analysis of the factors influencing biosecurity levels.

**Results:**

The results show that the greater the cost of biosecurity invested, the greater the level of biosecurity. Male farmers, educational attainment and participation in technical training had a significant positive effect on biosecurity levels. In addition, biosecurity levels first decreased and then increased as the scale of farming increased. The study also found that the more people in the household engaged in farming, the higher the biosecurity level of the farm. And farms that joined cooperatives had higher levels of biosecurity.

**Conclusion:**

The rank sum ratio method can evaluate the index system, so as to obtain a comprehensive index RSR value that can be compared, and the operation steps are simple and effective. By taking measures such as improving the comprehensive quality of farmers and increasing the investment in human, material and financial resources for biosecurity, the biosecurity level of farms can be effectively improved and animal diseases can be effectively prevented and controlled.

**Supplementary Information:**

The online version contains supplementary material available at 10.1186/s12917-023-03827-6.

## Background

Starting in August 2018, China reported several African swine fever that spread to many provinces in a few months, killing a large number of pigs, which had a significant impact on the pork industry. The transportation of live pigs was blocked, resulting in an imbalanced pork supply in some areas and great fluctuations in the price of pork. The sudden outbreak of African swine fever has had a great impact on farms, leading to limited productivity and heavy economic loss [1].

To prevent and control the spread of African swine fever in the country, the Chinese government issued a series of policies. For example, the General Office of the State Council issued Opinions on Strengthening the Prevention and Control of African Swine Fever [2]. And the Ministry of Agriculture and Rural Affairs issued the Emergency Implementation Plan for African Swine Fever (5th Edition) [3]. However, during the implementation of these policies, farmers discovered new cases of African swine fever and sold the infected pigs quickly at low prices. Farmers lied to the government, and the government’s regulatory functions had little impact on the situation. The prevention and control of the African swine fever epidemic should start from the source by improving the awareness of farmers on the prevention and control, giving incentives to the responsible farm owners, building biosecurity barriers on the farms, and strengthening relevant biosecurity measures. Most farm owners lack experience in and technical knowledge on the prevention and control of African swine fever virus, take inadequate prevention and control measures, and have low biosecurity levels in their breeding environments.

The frequent occurrence and difficult control of major animal diseases highlight the importance of animal health and its relationship to biosecurity [4]. The Food and Agriculture Organization of the United Nations (FAO) reports that biosecurity is directly related to agricultural sustainability, food safety, and environmental protection [5]. Biosecurity is also important to avoid the spread of diseases [6]. Preventing diseases through biosecurity measures improves the overall health of animal production [7]; a higher level of biosecurity will lead to the improvement of animal health and productivity and reduce the use of antibiotics and the frequency of antimicrobial treatment [8]. Epidemic-prone diseases are widely contagious, and biosecurity measures can reduce the risk of large numbers of livestock and poultry becoming infected with pathogens. However, differences in production scale, biosecurity standards, production input, and sales practices among pig farms affect the potential risk of disease transmission and lead to great differences in the ability of those farms to take preventive measures [9, 10]. At present, China's biosecurity situation is not optimistic, and some farms' biosecurity situation is worrying. The outbreak of African swine fever has caused a huge impact on them, with limited productivity and heavy economic losses. Therefore, it is necessary to understand and grasp the current situation of biosecurity of pig farms, and to explore influencing factors of biosecurity is helpful to improve the biosecurity level of farms.

The research on farm biosecurity in China has focused on the following two areas. The first is the assessment of the level of biosecurity prevention and control on farms and their weaknesses. Jia et al. interviewed 87 yellow catfish farmers in Guangdong and Zhejiang Province and developed a KAP index to assess farm-level biosecurity. The results showed that farmers scored higher on biosecurity attitudes than practice scores [11]. By combing through China's farm veterinary policies and regulations, Wei et al. assessed the weaknesses of the farm biosecurity infrastructure and institutional base [12]. The second is the preventive and control measures adopted by farmers and their influencing factors. Li et al. conducted a questionnaire survey of 153 pig farmers in Guangdong Province to study farm feeding, management and biosecurity measures, used logistic regression to identify risk factors and found that human exposure, poultry, wild birds and other animals on pig farms increased the risk of swine flu infection on farms [13]. Cui et al. investigated biosecurity measures implemented on poultry farms in Jiangsu and Anhui Provinces. The results of the multiple regression showed that farm size, stable cooperation with enterprises, etc. could influence the adoption of biosecurity practices [14].

A review of the existing literature on farm biosecurity in China shows that, on the one hand, most of the studies only focus on farms in a particular region or two or three provinces, and the samples are not representative; on the other hand, studies have either assessed the biosecurity of farms or investigated the factors influencing the adoption of biosecurity measures. Fewer studies have included both aspects. In the present study, survey data from Hebei, Liaoning, Jilin, Jiangsu, Sichuan, and Hubei provinces were used to evaluate the biosecurity levels of farms (households) using the rank sum ratio comprehensive evaluation method and then the influencing factors of biosecurity were analysed to provide empirical evidence for improving the biosecurity levels of farms and providing recommendations on disease prevention and control.

We believe that our study is a valuable addition for the biosecurity research domain. Some studies highlighted the significance of biosecurity in the prevention and control of major animal diseases and the influencing factors of biosecurity, but few studies have used quantitative analysis methods to empirically analyse the biosecurity levels in pig farms in China and relevant influencing factors. This study fills this gap in the literature. Further, previous studies on the assessment of the biosecurity level of pig farms are mostly based on the subjective evaluation method, which is generally assessed by the expert scoring method, and its objectivity is more insufficient. In this study, we constructed a farm biosecurity evaluation index system and used the rank sum ratio scoring method for the first time in the field of biosecurity, which is more operational and credible.

## Materials and methods

### Data and data management

The data used in the analyses were collected by the members of the research group on animal disease prevention and control of the Institute of Agricultural Economics and Development, Chinese Academy of Agricultural Sciences. This work was conducted under the research and application of the development model and benefit evaluation system of pig industry to improve quality and efficiency, transformation and upgrading project. The survey was conducted in the third quarter of 2020, and collected data on pig breeding and disease prevention and control in seven provinces (municipalities) of Sichuan, Hubei, Jiangsu, Tianjin, Liaoning, Jilin and Hebei. Among them, Tianjin was included as an infected area in the Northern China research site. The selection of provinces and municipalities for investigation is based on the following methods. Rank the number of slaughter fattened hogs in each province from high to low in 2018. The number of slaughter fattened hogs of the top 18 provinces was 629,858 thousand head, reaching 90.78% of the country (693,825 thousand head), which basically represents the pig production capacity in China; then, these 18 provinces are divided into 6 grades, each grade represents a different output level, and each grade selects a province as the representative of the corresponding production level. At the same time, considering the geographical differences of pig breeding, the provinces to be investigated are selected according to the six geographical divisions of China, among which the northwest region is not included in 18 provinces, and the remaining five regions are considered as a whole. See Table 1 for details of the selection of provinces and regions for investigation. In addition, in order to fully grasp the situation of African swine fever epidemic and non-epidemic areas, one endemic county and one non-endemic county were selected in each province. The specific distribution of the survey sample area is shown in Table 2. The survey adopted a combination of field interviews and questionnaires. A total of 267 questionnaires were collected by interviewing 267 farmers, and 246 valid samples were obtained, excluding those with missing data and abnormal samples. The survey respondents were pig farm owners. The questionnaire included the basic characteristics of pig farmers (e.g., age, sex, education, and breeding years), production and operation information of the farms (e.g., scale, type, and breeding mode), and biosecurity measures adopted by the farms, including isolation, disinfection, and personnel management.

**Table 1:** Number of slaughter fattened hogs by provinces in 2018

Rank	Province	Slaughter fattened hogs (thousand head)	Region
**1**	**Sichuan**	**66383**	**Southwest**
2	Henan	64024	Central South
3	Hunan	59937	Central South
4	Shandong	50823	East China
**5**	**Hubei**	**43635**	**Central South**
6	Yunnan	38505	Southwest
7	Guangdong	37574	Central South
**8**	**Hebei**	**37096**	**North China**
9	Guangxi	34658	Central South
10	Jiangxi	31240	East China
11	Anhui	28374	East China
**12**	**Jiangsu**	**26809**	**East China**
**13**	**Liaoning**	**24958**	**Northeast**
14	Heilongjiang	19644	Northeast
15	Guizhou	18699	Southwest
16	Chongqing	17582	Southwest
**17**	**Jilin**	**15704**	**Northeast**
18	Fujian	14213	East China
**19**	**Tianjin**	**2786**	**North China**

**Table 2:** Distribution of survey sample areas

Region	Province (Municipality)	Epidemic Area	Non-Epid emic Area	Sample Size	Proportion
North China	Hebei Province	Baoding (Anguo)	Luannan County, Ningjin County	27	10.98%
	Tianjin	Ninghe District	/	33	13.41%
Northeast	Jilin Province	Meihekou County, Liuhe County	Changchun (Nong ‘an, Dehui)	33	13.41%
	Liaoning Province	Jinzhou (Linghai)	/	31	12.60%
Central South	Hubei Province	Huanggang (Tuanfeng)	Huanggang (Xishui)	35	14.23%
East China	Jiangsu Province	Suqian (Muyang)	Yancheng (Funing)	47	19.11%
Southwest	Sichuan Province	Chengdu (Jintang)	Bazhong (Siyang)	40	16.26%
Sum				246	100%

### Data manipulation (pre-analysis)

#### Basic characteristics of survey sample distribution

Sample selection was performed based on the basic characteristics of pig farmers. Among the respondents, male pig farmers accounted for 82.93% of the total sample; the 45–54-year-old group accounted for half of the total; and the > 45-year-old group accounted for 74.8%; most farmers were middle-aged or elderly males (Table 3). Junior high school and senior high school graduates accounted for 89.84%. The distribution of the number of breeding years was relatively uniform. The group that bred pigs for 6–10 years was the largest (28.05%). In total, 78.86% of farmers received technical training, and only 20% did not receive technical training. Moreover, 92.28% of farmers' breeding income accounts for more than 50% of the total family income, among which 60.57% of farmers' production mode is self-breeding, followed by commercial pig fattening, accounting for 26.42%. There are 26 farms organized as cooperatives, accounting for 10.57%.

**Table 3:** Basic characteristics of survey sample distribution

Variable	Classification	Sample Size	Proportion/%	Variable	Classification	Sample Size	Proportion/%
Gender	Man	204	82.93	Technical training	Accepted	194	78.86
	Woman	42	17.07		Not accepted	52	21.14
Age	< 35 yr old	19	7.72	Breeding years	1–5 yr	53	21.54
	35–44 yr old	43	17.48		6–10 yr	69	28.05
	45–54 yr old	123	50		1–15 yr	55	22.36
	55–64 yr old	50	20.33		16–20 years	42	17.07
	> 65 yr old	12	4.47		> 20 years	28	10.98
Years of education	0–5 yr	12	4.88	Proportion of pig raising income to total family income	Under 25%	6	2.44
	6–10 yr	149	60.57		25–49%	13	5.28
	11–15 yr	72	29.27		50–74%	38	15.45
	16–20 yr	14	5.28		74% above	189	76.83
Production organization form	Cooperative farms	195	79.27	Level of operation scale	Less than 100 heads	81	32.93
	Others	51	20.73		100–499 head	62	25.20
Breeding mode	Self-breeding	170	69.11		500–999 head	33	13.41
	Commercial pig fattening	65	26.42		1000–4999 heads	61	24.80
	Piglet breeding	11	4.47		Over 5000 heads	9	3.66

#### Internal and external biosecurity of farms

Overall, 66.67% of the pig farms were more than 300 m away from the other farms, 67.89% of farms were more than 300 m away from the residential areas, and 19.51% of the farms were less than 300 m away from traffic trunk lines (Table 4). According to these three indicators, more than 60% of the farms in the survey sample met the ‘Technical Guide for Normalization Prevention and Control of African Swine Fever (Trial Edition)’ [15]. The technical guide provides that the site should be not less than 300 m away from the main traffic lines, and not less than 300 m away from residential areas and other livestock farms. In addition, 72.76% of farms were surrounded by natural isolation barriers, such as rivers, mountains, and farmland.

**Table 4:** Internal and external biosecurity of farms

Variable	Classification	Sample size	Proportion/%	Variable	Classification	Sample Size	Proportion/%
Distance from other farms	[0,0.3) km	82	33.33	Distance from residential area	[0,0.3) km	79	32.11
	[0.3,3) km	102	41.47		[0.3,3) km	144	58.54
	[3,10) km	51	20.73		[3,10) km	21	8.54
	[10,100] km	11	4.47		[10,35] km	2	0.81
Distance from traffic trunk line	[0,0.3) km	48	19.51	Fattening pigs all in and all out	Yes	83	33.74
	[0.3,3) km	141	57.32		No	163	66.26
	[3,10) km	45	18.29	Immune record	Built	216	87.80
	[10,20] km	12	4.88		Not under construction	30	12.20
Are there natural barriers around the farm (rivers, mountains, farmland)?	Yes	179	72.76	Does the farm manage sows, fattening pigs, and piglets by district?	Yes	200	81.30
	No	67	27.24		No	46	18.70
Is the means of transport special or exclusive?	Yes	177	71.95	Are clean roads and sewage roads separated in the field?	Yes	204	82.93
	No	69	28.05		No	42	17.07
Has the water source been tested?	Yes	109	44.31	Is drinking water disinfected?	Yes	90	36.59
	No	137	55.69		No	156	63.41

In terms of internal biosecurity, only 33.74% of the farms were engaged in all-in and all-out management of fattening pigs, which indicates that most farmers buy and sell fattening pigs in batches. In total, 81.30% of the farms bred sows, fattening pigs, and piglets in separate columns, and 18.70% of the farms were not managed by districts. Moreover, 71.95% of the farm transportation tools were used exclusively; 87.80% of the farms had immunization records; 82.93% were separated from sewage channels. More than half of the farms did not test or disinfect the drinking water sources of the pigs.

### Study 1. Construction and evaluation of biosecurity prevention and control system in pig farms

#### Construction of biosecurity prevention and control system in pig farms

The farm biosecurity system constructed in this study is based on the Chinese industry standards for pig breeding: specification for facilities and equipment configuration of large scale pig raising (NY/T 4254–2022) [16] and standardization farm–Swine (NY/T 2661–2014) [17], and references to relevant documents such as the United States Department of Agriculture (USDA)’s biosecurity guide for pork producers and the outdoor pig farm biosecurity evaluation system [18], the Biocheck.UGent™ biosecurity scoring system used by European Union countries, and the pig farm biosecurity technical specifications [19]. In this study, a biosecurity system was constructed considering both external and internal biosecurity measures. External biosecurity measures include site selection, biological control, material monitoring, and personnel and vehicle management, whereas internal biosecurity measures include cleaning and disinfection, disease prevention and control, feed and drinking water, pig herd management, and infrastructure allocation. The constructed biosecurity evaluation system primarily consists of two first-level indicators and nine second-level indicators of external and internal biosecurity; external biosecurity includes four indicators, and internal biosecurity includes five indicators, with a total of 24 third-level indicators (Table 5). The observed original data of all on-farm biosecurity indicators can be found in Additional file 1.

**Table 5:** Evaluation index system for biosecurity prevention and control on farms

Primary Index	Secondary Index	Three-Level Index	Sample size	
			**YES**	**NO**
External biosafety	Site selection	Distance (km) from nearest farm	See Table 4	
		Distance (km) from nearest residential area		
		Distance (km) from main traffic arteries		
		Are there natural barriers around the farm (rivers, mountains, farmland)?	179	67
	Biological control	Are there devices to prevent birds from entering the farm?	145	101
		Are there devices to prevent rodents from entering the farm?	163	83
		Are there devices to prevent insects from entering the farm? (such as ticks, mosquitoes, and flies)	151	95
	Material monitoring	Will feed be disinfected when it comes into play?	193	53
		Is the introduction of frozen semen tested for pathogens?	32	214
	And personnel and vehicle management	Can external personnel and vehicles freely enter the farm?	22	224
		Are there vehicles entering the decontamination site?	205	41
		Is the means of transport special or exclusive?	176	70
Internal biosafety	Cleaning and disinfection	Road disinfection frequency	See Additional file 1	
		Disinfection frequency of pen house		
		Is there any decontamination operation in the workers’ approach area? (bathing, disinfecting, changing clothes and boots, etc.)	230	16
	Epidemic prevention and control	Does the farm have an immunization record?	216	30
		Is there a resident veterinarian?	85	161
	Feed and drinking water	Whether feed uses kitchen waste (swill)	7	239
		Whether the water source is detected	109	137
		Whether drinking water is disinfected	91	155
	Pig herd management	Are fattening pigs all in and out?	82	164
		Are different pig groups managed by districts?	201	45
	Infrastructure allocation	Are clean roads and sewage roads separated in the field?	204	42
		Is there a transit site in the inner and outer sites?	173	73

#### Assessing biosecurity level of pig farms

To construct an evaluation index system for farm biosecurity, choosing the appropriate evaluation method is the next step. In this study, the rank sum ratio (RSR) method was used to evaluate the level of biosecurity on farms.

The RSR method is based on a nonparametric statistical method, which transforms the rank by index (column) and the number of groups (row) and then analyses the distribution of RSR by applying parameter analysis [20]. The RSR is used for evaluating the levels of multiple indicators. The RSR value ranges between 0 and 1 and continuous. Generally, the larger the value, the better the evaluation.

The calculation method can be described as follows:

-*Construct the evaluation matrix*. The evaluation matrix is an n*m matrix composed of n evaluation objects and m evaluation indices. The original data table is as follows, where ${\mathrm{x}}_{\mathrm{ij}}$ indicates the value of the jth evaluation index of the ith sample.

$$\mathrm{X}=\left(\begin{array}{ccc}{\mathrm{x}}_{11}& \cdots & {\mathrm{x}}_{1\mathrm{m}}\\ \vdots & \ddots & \vdots \\ {\mathrm{x}}_{\mathrm{n}1}& \cdots & {\mathrm{x}}_{\mathrm{nm}}\end{array}\right)$$

-*Rank arrangement*. There are two methods for ranking: the whole method and the non-whole method. In the whole rank method, high-quality indicators are ranked from small to large, low-quality indicators are ranked from large to small, and those with the same index data take the average value. Using the size of each specific evaluation index, rank R is obtained, and the original evaluation index value is replaced by rank R. According to the ranking results, the rank data matrix of each index can be established.
  $$\mathrm{R}=\left(\begin{array}{ccc}{\mathrm{R}}_{11}& \cdots & {\mathrm{R}}_{1\mathrm{m}}\\ \vdots & \ddots & \vdots \\ {\mathrm{R}}_{\mathrm{n}1}& \cdots & {\mathrm{R}}_{\mathrm{nm}}\end{array}\right)$$

Next, the RSR is calculated using Eq. (1), where n is the number of evaluation objects, m is the number of evaluation indices, and ${\mathrm{R}}_{\mathrm{ij}}$ is the rank of the ith evaluation object and the jth evaluation index.

1 $${\mathrm{RSR}}_{\mathrm{i}}=\frac{{\sum }_{\mathrm{j}=1}^{\mathrm{m}}{\mathrm{R}}_{\mathrm{ij}}}{\mathrm{n}*\mathrm{m}}$$

The RSR values are sorted and graded. According to the best grading principle, the evaluation objects are graded and classified. The number of steps is determined by the researcher according to the actual situation. In this paper, the level of biosecurity prevention and control in pig farms is divided into five grades. The RSR value representing the biosecurity level of a farm is calculated and used as the explained variable to empirically analyse the factors affecting biosecurity.

### Study 2. Empirical analysis of the influencing factors of biosecurity level in pig farms

#### Model Building

##### Multiple linear regression model

In the following multiple linear regression model,${\mathrm{RSR}}_{\mathrm{i}}$ indicates the biosecurity level of the ith farm, ${\mathrm{Z}}_{\mathrm{i}}$ is the main characteristics of farmers, including gender, age, years of education, breeding years, and whether one has received technical training; ${\mathrm{M}}_{\mathrm{i}}$ is the biosecurity cost; ${\mathrm{V}}_{\mathrm{i}}$ is the scale of farming, depending on whether ${\mathrm{B}}_{\mathrm{i}}$ is a cooperative farm or not; ${\mathrm{N}}_{\mathrm{i}}$ is the number of people engaged in farming in the family; ${\mathrm{R}}_{\mathrm{i}}$ is the proportion of farming income in total household income; ${\mathrm{S}}_{\mathrm{i}}$ is the dummy variable for the production mode; and PROV is the dummy variable for provinces, in which ${\upvarepsilon }_{\mathrm{i}}$ is random disturbance items.

2 $${\mathrm{RSR}}_{\mathrm{i}}=\mathrm{f}\left({{\mathrm{Z}}_{\mathrm{i}},{\mathrm{M}}_{\mathrm{i}},{\mathrm{V}}_{\mathrm{i}},{\mathrm{B}}_{\mathrm{i}},{\mathrm{N}}_{\mathrm{i}},{\mathrm{R}}_{\mathrm{i}},\mathrm{S}}_{\mathrm{i}},\mathrm{PROV}\right)+{\upvarepsilon }_{\mathrm{i}},$$

##### Tobit model

To further test the influencing factors of biosecurity level, the Tobit model was used for the robustness test. As the RSR value for the biosecurity level ranges from 0 to 1, which is a limited dependent variable, this value can be estimated by using the merge regression Tobit model. In the following Tobit model, ${\mathrm{y}}_{\mathrm{it}}^{*}$ is the horizontal vector of farm biosecurity, ${\upbeta }_{0}$ is the intercept vector, $\upbeta$ is the parameter vector to be estimated, ${\mathrm{x}}_{\mathrm{it}}$ is the explanatory variable vector, ${\upvarepsilon }_{\mathrm{it}}$ is the random disturbance term, and ${\mathrm{y}}_{\mathrm{it}}$ is the limited explanatory variable.

3 $$\begin{array}{c}{\mathrm{y}}_{\mathrm{it}}^{*}={\upbeta }_{0}+\upbeta {\mathrm{x}}_{\mathrm{it}}+{\upvarepsilon }_{\mathrm{it}},\\ {\mathrm{y}}_{\mathrm{it}}=\left\{\begin{array}{c}0,{\mathrm{y}}_{\mathrm{it}}^{*}\le 0\\ {\mathrm{y}}_{\mathrm{it}}^{*},0<{\mathrm{y}}_{\mathrm{it}}^{*}<1\\ 1,{\mathrm{y}}_{\mathrm{it}}^{*}\ge 1\end{array}\right.,\end{array}$$

#### Variable selection

##### Explained variables

The explained variable is the biosecurity level. Using the previously constructed evaluation system for the biosecurity of pig farms, the RSR value of the biosecurity level of the farm is obtained as the explained variable by using the RSR comprehensive evaluation method.

##### Explanatory variables

Explanatory variables include the basic characteristics of farmers, production and operation characteristics of farms, biosecurity cost as the core explanatory variable, and the province dummy variable. The basic characteristics of farmers include gender, age, years of education, and whether they have received technical training. The characteristics of production and operation include breeding years, degree of specialization, breeding scale, the number of farmers, production organization form, and production mode. Control variables also include ‘Whether ASF has occurred’ and ‘cognition on the ASF’. Cognition on blocking difficulty of African swine fever is divided into 5 classes. The class from 1 to 5 represents very little to very much difficulty in blocking.

Farmers are one of the important factors of production, and the number of farmers is the embodiment of labor force. The years of education and technical training can reflect the quality of breeders. Within a reasonable range, the improvement of farmers' quality will have a positive impact on the prevention and control of epidemic diseases, which is mainly reflected in their awareness of prevention and enthusiasm for taking measures when infected with epidemic diseases.

Breeding years are the years that farmers have been engaged in the pig industry, which mainly reflects the breeding experience of pig farmers. For example, farmers with longer breeding years have more experience in breeding decision-making and disease prevention than those with shorter breeding years, so it may play a positive role in improving the biosecurity level of farms. The degree of specialization refers to the degree of pig farmers' dependence on pig breeding, which is measured by the proportion of pig raising income to total family income. Breeding scale refers to the production level of a farm in a certain period and under specific conditions, and is an index to measure the breeding capacity of a farm. The scale of breeding is measured by the number of fattening pigs and sows in stock. There are three main production modes, including self-breeding, piglet breeding and commercial pig fattening.

Biosecurity cost, as a representation of biosecurity input, has a direct impact on the biosecurity level of farms, mainly including disinfection cost, treatment cost, veterinary cost, fee for adding drugs to feed and drinking water, etc. The descriptive statistics of all variables are shown in Table 6. See Additional file 3 for a detailed raw data list of all variables.

**Table 6:** Descriptive statistics of variables

Variables	Mean	Standard Deviation	Minimum	Maximum
**Explained variable**				
Biosecurity prevention and control level (RSR)	0.50	0.09	0.26	0.67
**Explanatory variable**				
Biosecurity cost	28,725.30	57,694.25	0.40	409,240.50
The basic characteristics of farmers				
Gender (male = 1, female = 0)	0.83	0.38	0.00	1.00
Age	49.10	8.94	26.00	77.00
Years of education	9.69	3.14	0.00	20.00
Have you received technical training in pig breeding? (yes = 1, no = 0)	0.79	0.41	0.00	1.00
The characteristics of production and operation				
Breeding years	12.39	7.32	1.00	40.00
Degree of specialization	84.90	22.13	10.00	100.00
Breeding scale	990.34	2437.69	3.00	30,000.00
Number of farmers	2.07	1.12	1.00	9.00
Production organization form (cooperation farms = 1, others = 0)	0.79	0.41	0.00	1.00
Production mode (compared with piglet breeding)				
Self-rearing (yes = 1, no = 0)	0.69	0.46	0.00	1.00
Fattening of commercial pigs (yes = 1, no = 0)	0.26	0.44	0.00	1.00
Whether ASF has occurred (yes = 1, no = 0)	0.37	0.48	0.00	1.00
Cognition on the ASF (very small = 1, relatively small = 2, average = 3, relatively large = 4, very large = 5)	4.45	0.92	1.00	5.00

### Cross-statistical analysis of explained variables and main explained variables

The relationship between several factors such as farming mode, technical training, farming scale, biosecurity cost, and biosecurity level was analysed, and the statistical relationship between the main factors and biosecurity level was evaluated using variance analysis.

## Results

### Study 1. Construction and evaluation of biosecurity prevention and control system in pig farms

#### Biosecurity level (Scores)

The biosecurity level is divided into five grades, and the higher the grade, the higher the biosecurity level. The biosecurity level of 3% farms is Grade I, and the score range of Rank Sum Ratio of Grade I is 0–0.347. The RSR value of Grade II is between 0.347 and 0.45, and the biosecurity level of 59 farms (24%) is Grade II. The biosecurity level of 111 farms (45%) is Grade III, and the RSR value is between 0.45 and 0.552. The RSR value of Grade IV is between 0.552 and 0.655, and 59 farms are Grade IV. The biosecurity level of 4% farms is Grade V, and the RSR value is between 0.655–1. Detailed results of rank sum ratio analyses for biosecurity prevention and control levels in all pig farms can be found in Additional file 2.

### Study 2. Empirical analysis of the influencing factors of biosecurity level in pig farms

#### Cross-statistical analysis

Relationship between the farming mode and biosecurity level. To determine whether different breeding modes will lead to differences in biosecurity levels, variance analysis was carried out on three modes of self-breeding, fattening of commercial pigs, and piglet breeding (Table 7). The average biosecurity level of farms with the piglet breeding mode is 0.46. The biosecurity levels are higher for farms engaged in self-breeding and the fattening of commercial pigs. The results of the variance analysis showed significant differences in the biosecurity levels of farms engaged in different farming modes.

**Table 7:** Relationship between production mode and biosecurity level

Group	Biosecurity Level Mean	Standard Deviation	Variance Analysis	
			**Variance Ratio**	***Prob* > *F***
Self-breeding	0.50	0.09	3.13	0.045
Commercial pig fattening	0.52	0.09		
Piglet breeding	0.46	0.10		

#### Relationship between farmers’ participation in technical training and biosecurity level

For farmers who received technical training, the average value of biosecurity level in their farms was 0.52, which was higher than the value among farmers who did not receive training. According to the results of the variance analysis (Table 8), the biosecurity level of farms whose farmers received technical training is significantly higher than that of farms whose farmers did not receive training, which indicates that technical training improves the biosecurity level.

**Table 8:** Relationship between technical training and biosecurity level

Group	Biosecurity level Mean	Standard Deviation	Variance Analysis	
			**Variance Ratio**	***Prob* > *F***
Receive technical training	0.52	0.08	32.31	0.000
No technical training	0.44	0.09		

#### Relationship between breeding scale and biosecurity level

According to the biosecurity score, the farms are divided into two groups with a cut-off point of 0.5. Those with a score greater than 0.5 are the first group, and the others are the second group. Statistical and variance analyses were performed on the breeding scales of two groups of farms with different biosecurity levels (Table 9). The breeding scale of one group of sample farms was significantly higher than that of the two groups of samples. The average breeding scale of the group with higher biosecurity level is 1421.75 m2, whereas that of the group with a lower biosecurity level is only 474.18 m2. The data show that the larger the farm scale, the higher the biosecurity level.

**Table 9:** Relationship between breeding scale and biosecurity level

Group	Breeding Scale Mean	Standard Deviation	Variance Analysis	
			**Variance Ratio**	***Prob* > *F***
Group 1 (RSR ≥ 0.5)	1421.75	3097.03	9.54	0.002
Group 2 (RSR < 0.5)	474.18	1062.95		

#### Relationship between biosecurity cost and biosecurity level

The average biosecurity cost of the group with a higher biosecurity level was nearly four times that of the group with a lower biosecurity level. The results of the variance analysis showed significant differences in biosecurity costs between farms with different biosecurity levels (Table 10). The higher the biosecurity cost of a farm, the higher that farm’s biosecurity level.

**Table 10:** Relationship between biosecurity cost and biosecurity level

Group	Biosecurity Cost Mean	Standard Deviation	Variance Analysis	
			**Variance Ratio**	***Prob* > *F***
Group 1 (RSR ≥ 0.5)	42.91	72.50	19.27	0.000
Group 2 (RSR < 0.5)	11.54	23.46		

#### Multiple linear regression estimation results and analysis

To test the multicollinearity among the explanatory variables, a variance expansion factor test was carried out. The results show that the maximum value for the variance expansion factor of the variables is 1.41, the minimum value is 1.04, and the average value is 1.19, which indicate no multicollinearity among variables.

Biosecurity cost has a positive impact on the level of biosecurity at the significance level of 1%, which indicates that farmers should increase their investments in the infrastructure construction of farm biosecurity and improve the level of farm biosecurity, which is consistent with the conclusions of Rodrigues (2019) that these types of investments help one better manage and maintain health [21].

From the perspective of the characteristics of pig farmers, the gender, and education levels of farm decision-makers affect the biosecurity levels of farms, which is consistent with previous research results [22]. This indicates that men are more concerned about biosecurity prevention and control than women, and that male farmers have a higher level of farm biosecurity. And the more educated farmers are more capable of acquiring knowledge and learning and are more likely to adopt biosecurity measures in a timely manner. Technical training plays a positive role in disease prevention and control at 0.1% significance level, which shows that farmers participate in technical training, improve their awareness of disease prevention and control, and actively take safety measures to prevent diseases [23, 24].

At the statistical significance level of 1%, the farming scale and square term of the farming scale have significant negative and positive effects on the level of biosecurity, respectively, indicating an u-shape relationship between farming scale and biosecurity level. When the scale becomes larger and does not exceed the turning point, the level of biosecurity decreases accordingly. On the one hand, due to the large scale, the required labor and material resources multiply. On the other hand, the larger the herd size, the greater the risk of transmitting pathogens within and between herds [25]. When the scale of farming reaches a turning point and expands to a larger scale, to prevent suffering huge financial losses, farmers pay more attention to the prevention and control of major animal diseases [26], which increases the biosecurity level.

At the significance level of 1%, the number of family members engaged in breeding has a positive impact on the level of biosecurity. With more family members involved in farming, there are more people available to help with various tasks on the farm, such as cleaning and disinfecting equipment and facilities, monitoring animal health, and implementing biosecurity protocols [27]. They may feel a stronger sense of ownership and responsibility for the farm's success. This can lead to a greater commitment to implementing and maintaining high levels of biosecurity, as everyone is invested in the health and well-being of the farm [28]. At the 1% significance level, the form of production organisation has a positive effect on the level of biosecurity on the farm, indicating that farms that join a cooperative have a higher level of biosecurity control due to the disease control services and guidance provided by the cooperative. No significant differences were observed between the biosecurity levels of farms in three production modes (self-breeding, commercial pig fattening, and piglet breeding). The detailed results of multiple regression empirical analysis are shown in Table 11.

**Table 11:** Empirical results for the influencing factors of the biosecurity levels on farms

	**Multiple Linear Regression**		**Tobit Regression**	
	**Coef**	**Std. Err**	**Coef**	**Std. Err**
Biosecurity cost (Logarithm)	0.0063**	(0.0023)	0.0063**	(0.0022)
The basic characteristics of farmers				
Gender	0.0226*	(0.0114)	0.0226*	(0.0110)
Age	-0.0007	(0.0006)	-0.0007	(0.0005)
Years of education	0.0046**	(0.0016)	0.0046**	(0.0015)
Technical training	0.0402***	(0.0113)	0.0402***	(0.0109)
Breeding years	-0.0007	(0.0007)	-0.0007	(0.0006)
The characteristics of production and operation				
Degree of specialization	-0.0002	(0.0002)	-0.0002	(0.0002)
Breeding scale (Logarithm)	-0.0441***	(0.0108)	-0.0441***	(0.0104)
Square term of breeding scale	0.0044***	(0.0011)	0.0044***	(0.0010)
Number of farmers	0.0115**	(0.0039)	0.0115**	(0.0038)
Production organization form	0.0342**	(0.0109)	0.0342**	(0.0106)
Production modes (compared with piglet breeding)				
Self-breeding	0.0227	(0.0209)	0.0227	(0.0202)
Commercial pig fattening	0.0251	(0.0221)	0.0251	(0.0213)
Whether ASF has occurred	0.0079	(0.0095)	0.0079	(0.0092)
Cognition on blocking difficulty	-0.0025	(0.0048)	-0.0025	(0.0046)
Area dummy variable	Control		Control	
_cons	0.4744***	(0.0616)	0.4744***	(0.0594)

Standard errors in parentheses

^*^ *p* < 0.05, ** *p* < 0.01, *** *p* < 0.001

#### Robustness test

The results of the Tobit model show that the chi-square test rejected the original assumption of zero variable model at the significance level of 1%, which indicates a good model fit (Table 11). Comparison of the regression results of the Tobit model with those of the multiple linear regression model shows that the significance level is consistent with the estimation coefficient, which demonstrates the robustness of the results.

## Discussion

The term biosecurity is used to describe all measures that can reduce the entry and spread of pathogens on farms [29]. World Health Organization (2010) defines biosecurity as a comprehensive method to manage human, animal, and plant life and health risks [30]. In the Animal Production and Health Report issued by the FAO, biosecurity is defined as a series of effective preventive measures to stop pathogens from entering herds or farms and prevent diseases from spreading to uninfected animals in herds or other farms when pathogens already exist [31]. For pig herds, biosecurity refers to preventing the introduction and spread of viruses, bacteria and other infectious factors. [32].

When analysing the farm biosecurity, previous research mainly considers the following factors: the locations of farms, disinfection, vehicles and feeds, workers and visitors, waste and carcass disposal, wild animals, and pests, and whether all-in and all-out management is taken for herds [10, 33, 34]. Alarcón et al. [4] summarized the most common external biosecurity measures, including the introduction of breeding, quarantining, semen use, personnel and vehicles, animal transportation, farm location, and feed and water, as well as internal biosecurity, which involves measures related to herd management, general sanitation of the infrastructure, cleaning and disinfection, and personnel management. The BioCheck.UGent™ scoring system (available at www.biocheck.ugent.be), developed by Laanen et al. [35], divides biosecurity into two parts: external and internal biosecurity. External biosecurity involves preventing pathogens from entering herds, whereas internal biosecurity reduces the spread of pathogens within herds. BioCheck.UGent™, which has been widely used to evaluate the biosecurity status of herds [7, 21], has 109 questions that are divided into six external and six internal biosecurity subcategories. The subcategories of external biosecurity are livestock and poultry transportation, feed, water and equipment, personnel and visitors, pest and bird control, and the surrounding environment and areas. The subcategories of internal biosecurity are disease management, childbirth and lactation, piglet breeding, fattening management, the use of biosecurity measures and equipment between compartments, cleaning, and disinfection.

The rank sum ratio (RSR) method was developed in 1988 by Tian Fengtiao [36], a famous health statistician in China, and has been widely applied since then. Yu et al. [37] applied the RSR method to the field of working-fluid selection for the first time by simply and effectively selecting the appropriate working fluid for a thermodynamic system. Tan et al. [38] used the weighted RSR method to identify statistically significant failure modes and the weakest failure modes of bridges. Wang et al. [39] used this method to create a feeding-practice index to evaluate the feeding-practice behaviours in rural areas of Lhasa, Tibet. This comprehensive evaluation method offers strong operability, convenience, and ease of use.

The application of biosecurity measures largely depends on farmers’ attitudes and understanding of infectious diseases and their prevention [40]. Farmers’ understanding of the importance of different biosecurity measures is a complicated process related to many factors and can be influenced by farm characteristics, measures implemented or taken by neighbouring farms, veterinary advice, and existing scientific and technical information [41]. Ribbens et al. [8] showed that the type and scale of a farm influence that farm’s biosecurity application degree. Casalet al. [41] reported that on more specialized farms, farmers pay more attention to disease prevention. Laanen et al. [35] noted that the larger the herd size, the higher the external biosecurity score, with a negative correlation between farmer experience and internal score; the younger the farmer, the higher the biosecurity level. Backhans et al. [42] showed that farm owners who are women or have less than 23 years of experience will have a higher biosecurity score. The authors also detected a positive correlation between education level and internal biosecurity score.

Some studies [43–45] highlighted the significance of biosecurity in the prevention and control of major animal diseases and the influencing factors of biosecurity, but few studies have used quantitative analysis methods to empirically analyse the biosecurity levels in pig farms in China and relevant influencing factors.

## Conclusions

Biosecurity measures on farms can effectively prevent and control animal diseases, improve the quality of livestock products, and ensure consistent meat supply. In this study, an evaluation system for farm biosecurity was constructed. Based on survey data from seven provinces (municipalities directly under the Central Government) in China, a comprehensive evaluation method of the rank sum ratio was used to obtain the biosecurity levels of the investigated farms, and the influencing factors of the biosecurity levels were empirically analysed. The results can be summarized as follows: (1) According to the survey results, the biosecurity levels on farms are not ideal. The site selection and distribution of some farms still do not meet the requirements, and the distance from residential areas, traffic arteries, and other farms is not sufficient to ensure compliance with epidemic prevention requirements. Some farm owners are not aware of the disinfection guidelines for drinking water, epidemic prevention records, zoning management, the use of all-in, all-out replacement schedules, and special transportation tools. (2) The empirical results show that farms where the farmer is male, better educated and technically trained have a higher level of biosecurity. The more people in the household engaged in farming, the higher the biosecurity level of the farm. As the size of the farm increases, the biosecurity level of the farm first decreases and then increases. However, the level of biosecurity on farms did not increase with the number of breeding years. The biosecurity level of farms that joined the co-operative was higher than those that did not. In addition, the higher the cost of biosecurity inputs, the higher the level of biosecurity on the farm. (3) The rank sum ratio method was used to evaluate the index system and provided a comprehensive index value that can be compared while using the operation steps that are simple and effective.

This study has the following implications: (1) The current requirements and regulations of farm biosecurity, the publicity and education, and reliance on farmers’ own consciousness and knowledge are not sufficient to form a strong epidemic prevention barrier. Government and social entities should promote publicity, education, and skill training to make up for the shortcomings of farmers’ lack of awareness, knowledge, and abilities. (2) The infrastructure is complete, the rules and regulations are sufficient, and farm construction meets the ecological requirements to build large ecological-scale farms. The epidemic prevention infrastructure and site selection are scientifically reasonable. These factors not only meet the relevant ecological requirements but also provide an effective barrier to prevent and control epidemic-prone diseases. (3) Decision-makers should establish a biosecurity system for farms, formulate a biosecurity standard approval system for pig farm construction, use administrative means to raise awareness of disease prevention and control among farmers, and build and equip biosecurity facilities.

### Supplementary Information

**Additional file 1.** The observed original data of all on-farm biosecurity indicators.

**Additional file 2.** Results of rank sum ratio analyses for biosecurity prevention and control levels in all pig farms.

**Additional file 3.** The detailed raw data list of all variables for empirical analysis.

## Data Availability

All data generated or analyzed during this study are included in this published article and its supplementary information files.
